# Halogenated indoles eradicate bacterial persister cells and biofilms

**DOI:** 10.1186/s13568-016-0297-6

**Published:** 2016-12-05

**Authors:** Jin-Hyung Lee, Yong-Guy Kim, Giyeon Gwon, Thomas K. Wood, Jintae Lee

**Affiliations:** 1 School of Chemical Engineering, Yeungnam University, Gyeongsan, 38541 Republic of Korea; 2 Department of Chemical Engineering, Pennsylvania State University, University Park, PA USA

**Keywords:** Antibiotic resistance, Biofilms, *Escherichia coli*, Indoles, Persisters, *Staphylococcus aureus*

## Abstract

The emergence of antibiotic resistance has necessitated new therapeutic approaches to combat recalcitrant bacterial infections. Persister cells, often found in biofilms, are metabolically dormant, and thus, are highly tolerant to all traditional antibiotics and represent a major drug resistance mechanism. In the present study, 36 diverse indole derivatives were investigated with the aim of identifying novel compounds that inhibit persisters and biofilm formation by Gram-negative *Escherichia coli* and Gram-positive *Staphylococcus aureus*. 5-Iodoindole and other halogenated indoles, 4-fluoroindole, 7-chloroindole, and 7-bromoindole, eradicated persister formation by *E. coli* and *S. aureus,* and 5-iodoindole most potently inhibited biofilm formation by the two bacteria. Unlike other antibiotics, 5-iodoindole did not induce persister cell formation, and 5-iodoindole inhibited the production of the immune-evasive carotenoid staphyloxanthin in *S. aureus*; hence, 5-iodoindole diminished the production of virulence factors in this strain. These results demonstrate halogenated indoles are potentially useful for controlling bacterial antibiotic resistance.

## Introduction

The long-term use of bactericidal and bacteriostatic antibiotics has generated multidrug resistant pathogens, such as, *Staphylococcus aureus* (Levy and Marshall 2004; Cegelski et al. 2008), and resulted in a public health crisis. Of the various mechanisms of antibiotic resistance, the formation of bacterial persisters and biofilms are interlinked and difficult to control (Lewis 2007, 2008). Therefore, a non-antibiotic strategy, which targets persisters and biofilms, is required to control bacterial infections.

Unlike drug resistant cells, which grow in the presence of antibiotics due to genetic changes (Lewis 2010), persister cells are metabolically dormant cells and highly tolerant of antibiotics without undergoing any genetic change (Wood et al. 2013; Keren et al. 2004), and account for a maximum of ~1% of cells in the stationary phase and in biofilms (Lewis 2007, 2008). However, little is known about the signals and pathways leading to persister formation, although toxin/antitoxin systems appear to constitute a primary mechanism, as they can be used to induce a state of dormancy (Lewis 2008; Wang and Wood 2011).

Bacterial biofilms are sessile microbial communities that attach to surfaces using self-produced extracellular polymeric substances, such as, polysaccharides, proteins, and nucleic acids. Due to their high antimicrobial tolerances, biofilms formed by pathogenic bacteria pose serious problems to human health, for example, pathogenic *P. aeruginosa*, *C. albicans*, and *E. coli* biofilms are believed to be causative agents of cystic fibrosis, prostatitis, periodontitis, and urinary catheter cystitis (Costerton et al. 1999). Biofilms protect significant numbers of persister cells from antibiotics and immune systems (Lewis 2008), and thus, biofilms and persisters are closely related. Furthermore, a significant amount of biofilm research has been undertaken with the aim of discovering non-toxic compounds that can inhibit biofilm formation without allowing bacteria to develop drug resistance.

Indole is produced by a large number of Gram-positive and Gram-negative bacterial species, including *E. coli* (Lee and Lee 2010), and as an interspecies and interkingdom signaling molecule, indole plays important roles in various bacterial phenotypes and even in eukaryotic immunity (Bansal et al. 2010; Lee et al. 2010; Vega et al. 2012; Erb et al. 2015). In particular, indole has been reported to modulate biofilm formation in various bacteria (Lee et al. 2007; Oh et al. 2012; Lee et al. 2015) and persister formation in *E. coli* (Vega et al. 2012, 2013; Hu et al. 2015; Kwan et al. 2015). Of note, functional groups on the indole moiety control biofilm formation (Lee et al. 2009), the production of virulence factors and antibiotic resistance in *Pseudomonas aeruginosa* (Lee et al. 2009, 2012), spore maturation in *Paenibacillus alvei* (Kim et al. 2011), and the production of immune-evasive staphyloxanthin in *S. aureus* (Lee et al. 2013). Therefore, because the demonstrated potential efficacies of indole derivatives against several bacteria, we reasoned that some indole derivatives would have greater anti-persister activities than indole itself.

In the present study, we investigated diverse indole derivatives in an attempt to identify halogenated indoles that inhibit persister formation and biofilm formation by Gram-negative *E. coli* and Gram-positive *S. aureus.*

## Materials and methods

### Bacterial strains, reagents and strain maintenance

**Fig. 1:**
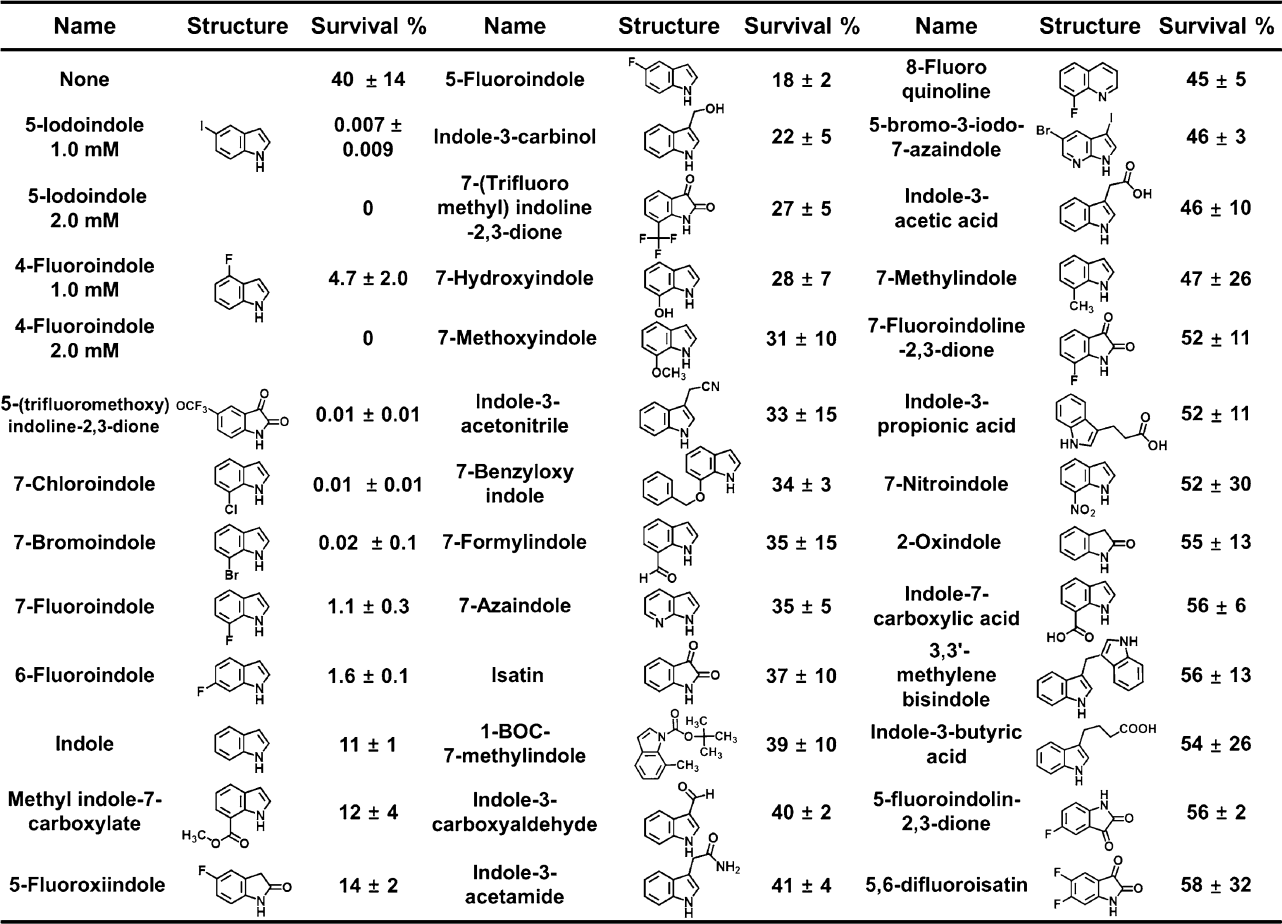
Indole derivatives kill *E. coli* persister cells. *Escherichia coli* K-12 BW25113 cells were exposed to rifampicin (100 μg mL^−1^) to induce persister cells. Indole or indole derivatives at 2 mM were then administered for 3 h at 37 °C and 250 rpm, and cell viabilities were determined. All indole compounds were dissolved in DMSO, and DMSO was used as the control (none). For the most active compounds, 5-iodoindole and 4-fluoroindole, two concentrations (1 and 2 mM) were tested. The experiment was performed in duplicate

The following bacterial strains were used in the study; *E. coli* K-12 BW25113, *E. coli* O157:H7 (86–24), methicillin-sensitive *S. aureus* ATCC 6538, methicillin-resistant *S. aureus* ATCC 33591 and ATCC BAA-1707, and *Pseudomonas aeruginosa* PAO1. *E. coli* BW25113 strain was chosen to compare the efficacy of indoles to the previous results of *E. coli* persister cells (Hu et al. 2015; Kwan et al. 2015). In addition, three human pathogens, such as Gram-negative *E. coli* O157:H7 and *P. aeruginosa* and Gram-positive *S. aureus* were investigated to understand the general efficacy of indoles in the eradication of persister cells. All experiments were conducted at 37 °C, and bacteria were cultured in Luria–Bertani (LB) medium. Indole, thirty-five commercially available indole derivatives (Fig. 1), and three antibiotics were purchased from Sigma-Aldrich (St. Louis, USA) or Combi-Blocks, Inc. (San Diego, USA). The chemical structures of these derivatives are shown in Fig. 1. All indole derivatives were dissolved in dimethyl sulfoxide (DMSO), which was also used as a control. Bacteria were initially streaked from −80 °C glycerol stock on LB plates and fresh single colonies were inoculated in LB (25 mL) in 250 mL flasks and cultured at 37 °C at 250 rpm. For cell growth measurements, optical densities with or without indoles were measured at 600 nm using a spectrophotometer (Optizen 2120UV, Mecasys, Korea). Experiments were performed using at least three independent cultures.

### Minimum inhibitory concentration (MIC) assay

The MICs of indoles for *E. coli* BW25113, *S. aureus* ATCC 6538, a methicillin-resistant *S. aureus* ATCC 33591, and *P. aeruginosa* PAO1 were determined by incubating freshly inoculated cultures in LB for 24 h containing varying concentrations of indoles and observing growth inhibition based on lack of turbidity. Results were confirmed by CFU counting. Experiments were performed using at least two independent cultures.

### Persister cell killing assay

Persister cell survival was determined by counting the number of colonies that grew on solid media after washing and serially diluting cells exposed to antibiotics, as previously described (Kwan et al. 2013). Briefly, overnight cultures (16 h) were diluted 1:1000 with fresh LB medium and grown to the desired turbidity (0.8 at 600 nm for the exponential phase and 3–4 at 600 nm for the mid-stationary phase) at 250 rpm. Since pretreatments of bacteriostatic rifampicin (Kwan et al. 2013) and bactericidal ampicillin and ciprofloxacin (Hu et al. 2015; Kwan et al. 2015) increased persister cell formation, the three antibiotics have been used in this study. In order to obtain antibiotic-induced persister cultures in buffered LB, cultures were exposed to rifampicin (100 μg mL^−1^), ampicillin (100 μg mL^−1^), or ciprofloxacin (0.5 μg mL^−1^) and incubated for 30 min at 37 °C. Cells were harvested by centrifugation at 4000 rpm for 14 min and washed with fresh LB (turbidity was controlled as 0.5). Cells (0.5 mL) were then transferred to micro-tubes and treated with or without indoles and incubated for 3 h at 37 °C at 250 rpm. DMSO was used as a control. Cell viabilities were determined by serially diluting cells with PBS buffer, plating 100 μL drops on LB agar, and counting colonies.

### Persister formation induced by antibiotics, indole, and 5-iodoindole

In order to test the effect of 5-iodoindole on persister formation, overnight cultures of *E. coli* BW25113 or *S. aureus* 6538 were diluted 1:1000 with 25 mL of LB and incubated at 37 °C with shaking at 250 rpm to a turbidity of 0.8 at 600 nm. Cells were then treated with indole (2 mM), 5-iodoindole (2 mM), ampicillin (100 μg mL^−1^) or rifampicin (100 μg mL^−1^) and incubated at 37 °C with shaking at 250 rpm for 0, 0.5, 1, or 3 h. Cell viabilities were determined by serial dilution in PBS buffer, plating 100 μL drops on LB agar, and counting colonies. DMSO was used as a negative control.

### Crystal-violet biofilm assay

Crystal-violet biofilm assays were performed in 96-well polystyrene plates (SPL Life Sciences, Korea), as previously described (Lee et al. 2011). Briefly, overnight cultures were inoculated into LB medium (total volume 300 μL) at an initial turbidity of 0.05 at 600 nm and cultured with or without indoles for 24 h without shaking at 37 °C. To quantify biofilm formation, cell cultures were washed three times with H_2_O to remove non-adherent cells. Biofilms were stained with 0.1% crystal violet for 20 min, rinsed three times with H_2_O, extracted with 95% ethanol, and absorbances were measured at 570 nm. Results are the averages of at least twelve replicate wells.

### Confocal laser scanning microscopy and COMSTAT analysis

*Escherichia coli* BW25113 and *S. aureus* cells were cultured in 96-well polystyrene plates (SPL Life Sciences, Korea) without shaking with or without 5-iodoindole. Biofilms were stained with carboxyfluorescein diacetate succinimidyl ester (Invitrogen, Molecular Probes, Inc., Eugene, OR, USA) (Weston and Parish 1990). Planktonic cells were removed by washing with PBS three times, and static biofilms were visualized by excitation using an Ar laser 488 nm (emission wavelengths 500–550 nm) under a confocal laser microscope (Nikon Eclipse Ti, Tokyo) using a 20× objective (Kim et al. 2012). Color confocal images were constructed using NIS-Elements C version 3.2 (Nikon eclipse). For each experiment, at least 10 random positions in two independent cultures were chosen for microscopic analysis.

To quantify biofilm formation, color confocal images (20 image stacks) were converted to gray scale using ImageJ. COMSTAT biofilm software (Heydorn et al. 2000) was used to determine biomasses (μm^3^ μm^−2^), mean thicknesses (μm), and substratum coverages (%). Thresholding value was fixed for all image stacks, and at least 4 positions and 20 planar images per position were analyzed.

### Staphyloxanthin assay

The production of *S. aureus* staphyloxanthin, which has a golden color, was assayed as previously described (Lee et al. 2013). Briefly, cells of a methicillin-sensitive *S. aureus* ATCC 6538 or of a methicillin-resistant *S. aureus* ATCC BAA-1707, were inoculated at 1:100 dilution in trypticase soy broth (2 mL) and incubated for 24 h with indole derivatives at 37 °C in 14 mL tubes at 250 rpm. Cells (1 mL) were then collected by centrifugation at 16,600×*g* for 10 min, and cell pellets were photographed to compare staphyloxanthin production.

### Transmission electron microscope (TEM) assay

Morphological changes of *E. coli* and *S. aureus* after antibiotic rifampicin and 5-iodoindole treatment were examined as previously described with some modification (Kim et al. 2011). As mentioned above, bacteria were exposed to rifampicin (100 μg mL^−1^) and incubated for 30 min at 37 °C, washed, and exposed to 5-iodoindole (2 mM) for 1 h at 37 °C and 250 rpm. Cells were then fixed initially with an aldehyde mixture of 50% glutaraldehyde and 35% formaldehyde, incubated at 4 °C overnight, collected by centrifugation, then fixed with 1% osmium tetroxide overnight at 4 °C, and washed three times. Cell pellets were mixed with 3% agarose and sliced to desired sizes, and slices were dehydrated and embedded in an Epon resin mixture (Hatfield, USA). Slices containing embedded cells were then sectioned using a MT-X ultramicrotome (Tucson, USA), loaded onto TEM copper grids, stained with uranyl acetate, and treated with lead citrate. Scanning electron microscopy was performed using a H-7600 electron microscope (Hitachi, Tokyo) at 80 keV.

### Total mRNA isolation

Total mRNA was used to study the effects of indole and 5-iodoindole on mRNA synthesis. Briefly, *E. coli* BW25113 was inoculated into 25 mL of LB medium in 250 mL shake flasks at a starting turbidity of 0.005 at 600 nm and grown to a turbidity of 0.8. Cultures were then exposed to rifampicin (100 μg mL^−1^) for 30 min at 37 °C with shaking at 250 rpm to create persister cells (Kwan et al. 2013), washed with fresh LB, and incubated in the presence or absence of indole or 5-iodoindole (2 mM) for 1 h. RNase inhibitor (RNAlater, Ambion, TX, USA) was added to prevent RNA degradation. Total RNA was isolated using a Qiagen RNeasy mini Kit (Valencia, CA, USA). At least two independent cultures were performed per experiment.

## Results

### Indoles kill *E. coli* persister cells

Since indole has been reported to modulate the survival of *E. coli* persister cells (Vega et al. 2012; Hu et al. 2015; Kwan et al. 2015), we investigated the abilities of indole and 35 commercially available indole derivatives at a concentration of 2.0 mM to reduce the survival of *E. coli* K-12 BW25113 persister cells. For initial screening, *E. coli* cells were treated with rifampicin (100 μg mL^−1^) to convert exponentially growing cells to persister cells (Kwan et al. 2013). As shown previously (Kwan et al. 2013), rifampicin pretreatment increased persister cell levels to 40 ± 14% (Fig. 1). After rifampicin treatment, indole at 2.0 mM reduced persister survival from 40 to 11%, which concurred with previous reports (Hu et al. 2015; Kwan et al. 2015). More importantly, indole derivatives demonstrated a wide range of inhibitory abilities to kill persister cells (Fig. 1). Of the tested compounds, five halogenated indoles, namely, 5-iodoindole, 4-fluoroindole, 5-(trifluoromethoxy)indoline-2,3-dione, 7-chloroindole, and 7-bromoindole reduced *E. coli* K-12 BW25113 persister survival by more than 2000-fold, whereas other indole derivatives had less impact on persister survival (Fig. 1). In particular, 5-iodoindole and 4-fluoroindole at 2.0 mM completely prevented persister survival, and at 1.0 mM, 5-iodoindole was more potent (0.007% survival) than 4-fluoroindole (4.7% survival). Accordingly, 5-iodoindole became the focus of further study.

**Fig. 2:**
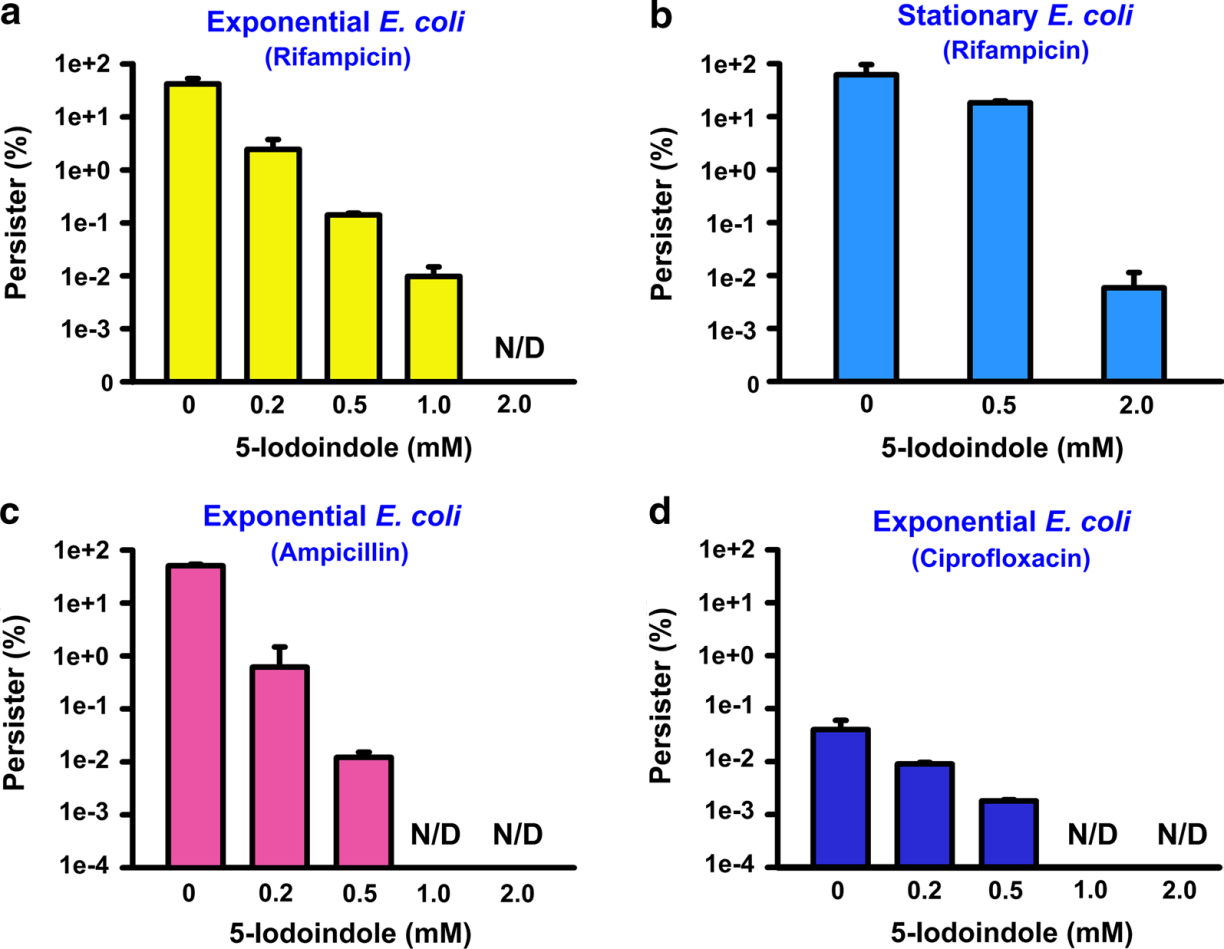
5-Iodoindole eradicates *E. coli* persister cells. *Escherichia coli* K-12 BW25113 cells in the exponential growth stage (**a**) or stationary growth stage (**b**) were exposed to rifampicin (100 μg mL^−1^) to induce persister cells, which were then treated with 5-iodoindole for 3 h at 37 °C and 250 rpm. Cell viabilities were determined. Ampicillin at 100 μg mL^−1^ (**c**) or ciprofloxacin at 0.5 μg mL^−1^ (**d**) were used to induce persister cells and then 5-iodoindole was treated. The experiment was performed in duplicate. *N/D* represents eradication below the limit of detection

Further persister assays showed 5-iodoindole dose-dependently reduced persister survival and that at a concentration of only 0.2 mM it reduced persister survival by 20-fold versus the non-treated control (Fig. 2a). Furthermore, 5-iodoindole effectively killed persister cells originally derived from stationary and exponential phase cells (Fig. 2b), and significantly reduced the survival of persisters pretreated with ampicillin and ciprofloxacin (Fig. 2c, d). Specifically, no persister cells (no colonies) were detected after treating ampicillin- or ciprofloxacin-generated persister cells with 5-iodoindole at concentrations >1.0 mM. Since ampicillin and ciprofloxacin are representative of two different classes of bactericidal antibiotics and because rifampicin is a bacteriostatic antibiotic that inhibits mRNA synthesis (Calvori et al. 1965), these findings suggest 5-iodoindole effectively kills persister cells generated by bactericidal antibiotics and bacteriostatic rifampicin.

### 5-Iodoindole eradicates the persister cells of human pathogens

**Fig. 3:**
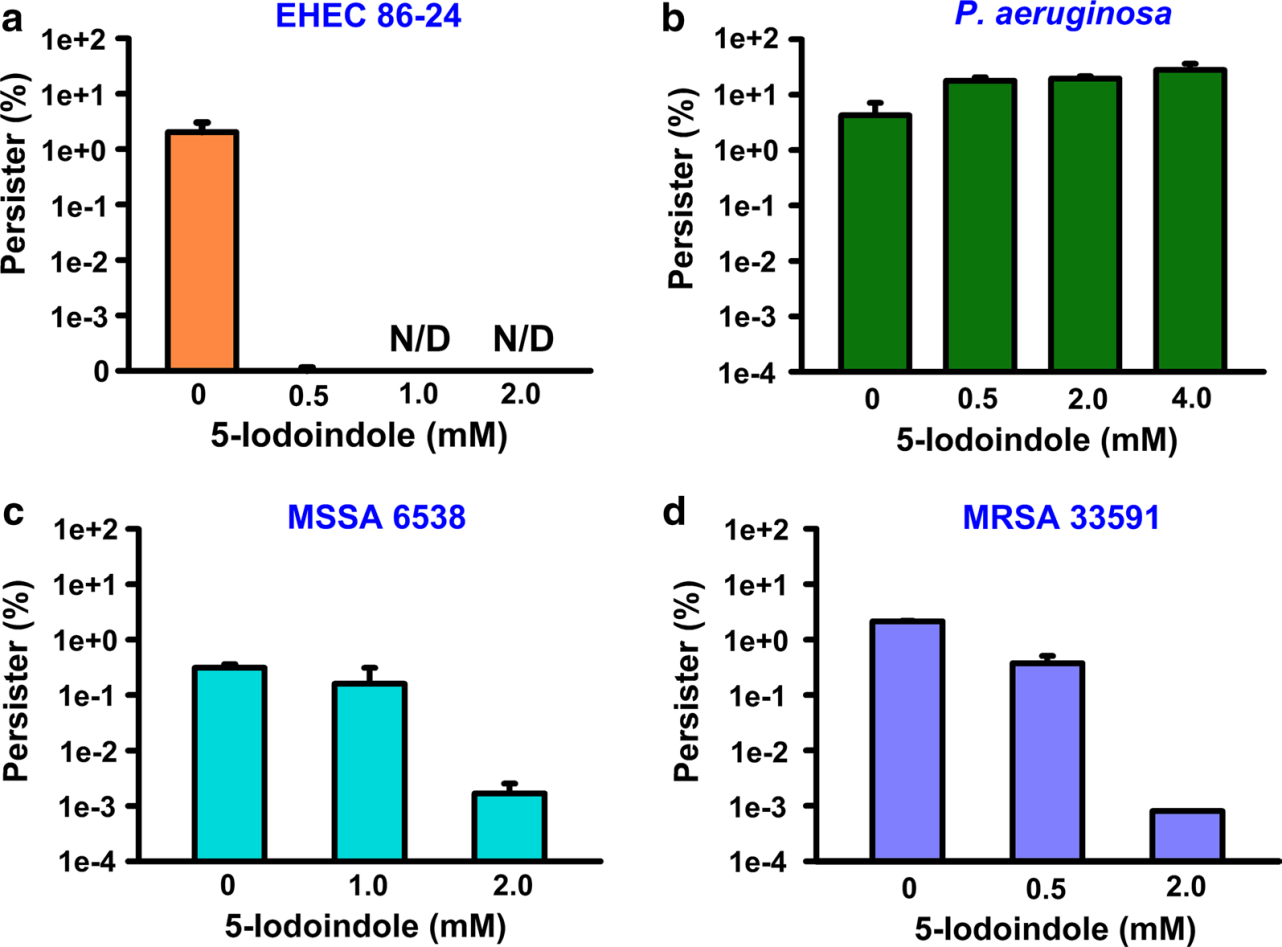
5-Iodoindole kills persister cells of human pathogens. *Escherichia coli* O157:H7 (EHEC, **a**), *P. aeruginosa* (**b**), methicillin-sensitive *S. aureus* (MSSA) 6538 (**c**), or a methicillin-resistant *S. aureus* (MRSA) 33591 (**d**) were exposed to rifampicin (100 μg mL^−1^), and then 5-iodoindole was treated for 3 h at 37 °C and 250 rpm. Cell viabilities were then determined. The experiments were performed in duplicate. *N/D* represents eradication below the limit of detection

The ability of 5-iodoindole to eradicate other human pathogens, that is, enterohemorrhagic *E. coli* O157:H7 (EHEC), methicillin-sensitive *S. aureus* ATCC 6538 (MSSA 6538), methicillin-resistant *S. aureus* ATCC 33591 (MRSA 33591), and *P. aeruginosa.* 5-Iodoindole was found to be effective against EHEC, that is, at 1.0 mM 5-iodoindole completely eradicated persister cells (Fig. 3a), and 5-iodoindole dose-dependently reduced MSSA 6538 and MRSA 33591 persister survival (Fig. 3c, d). However, 5-iodoindole at concentrations of up to 4 mM did not reduce *P. aeruginosa* persister survival (Fig. 3b).

The minimum inhibitory concentrations (MICs) of halogenated indoles were then measured since general antibiotic treatments were administered at least five times their MICs (Wood 2016) to minimize the survivals of potentially resistant cells. The MICs of 5-iodoindole were between 1.0 and 2.0 mM for *E. coli* and *S. aureus* strains, and the MICs of other indoles (4-fluoroindole, 7-chloroindole, and 7-bromoindole) fell in the range 2.0–3.0 mM (Table 1). Hence, 5-iodoindole at a concentration of 1× its MIC level markedly reduced *E. coli* K-12 and EHEC persister survival, and persister survival of two *S. aureus* strains, including MRSA. Accordingly, *E. coli* and *S. aureus* strains were the subjects of further study.

**Table 1:** MICs of the indoles examined in this study

Strain	Indole (mM)	5-Iodoindole (mM)	4-Fluoroindole (mM)	7-Chloroindole (mM)	7-Bromoindole (mM)
*E. coli* BW25113	5.0	1.5	2.0	2.0	2.0
*S. aureus* MSSA 6538	20	2.0	3.0	3.0	3.0
*S. aureus* MRSA 33591	20	1.0	3.0	3.0	3.0
*P. aeruginosa* PAO1	10	>50	5	10	>50

### 5-Iodoindole inhibits *E. coli* and *S. aureus* biofilm formation

**Fig. 4:**
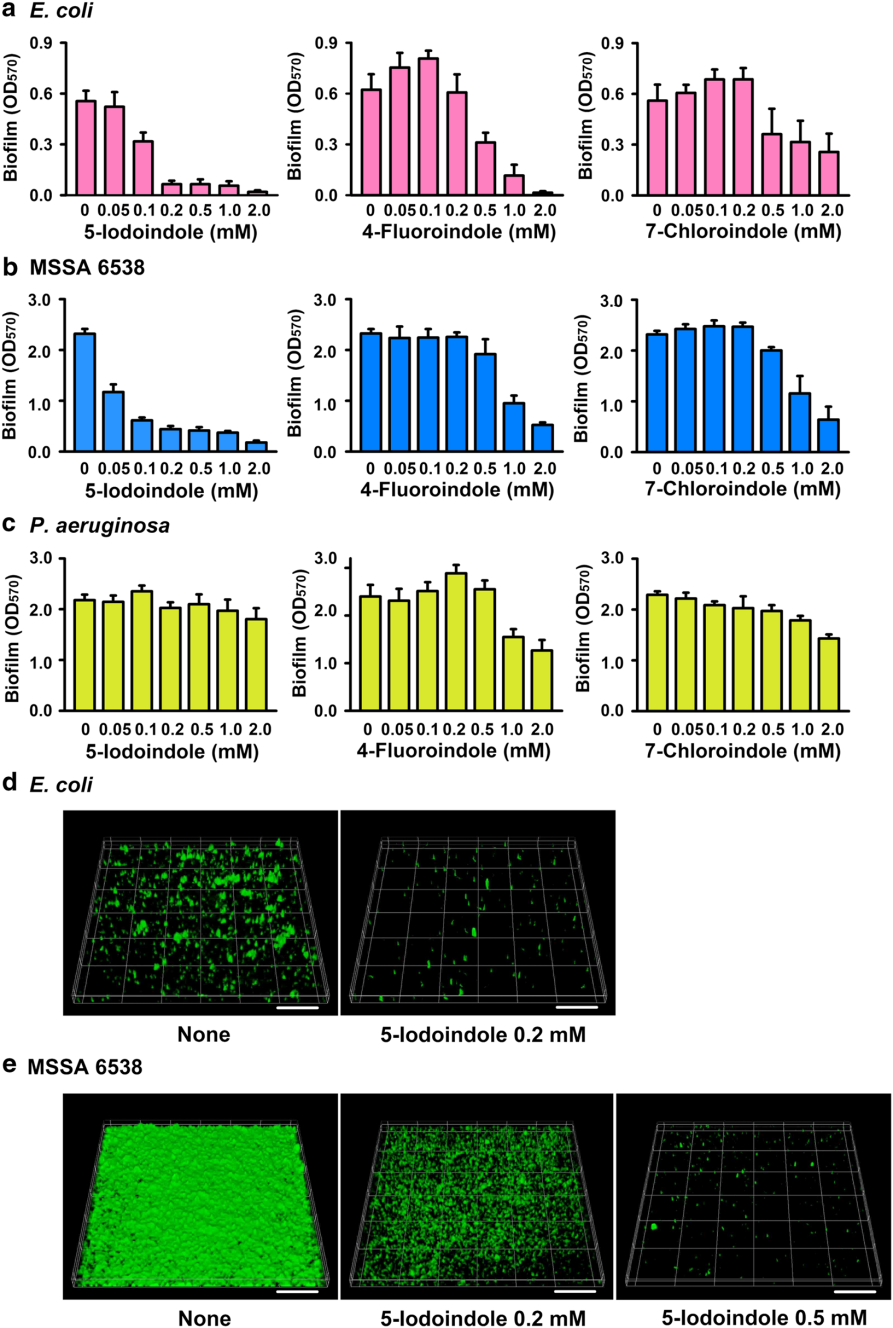
Substituted indoles reduce the biofilm formation of *E. coli* and *S. aureus. Escherichia coli* K-12 BW25113 (**a**), *S. aureus* (MSSA) 6538 (**b**), and *P. aeruginosa* (**c**) biofilm formation was quantified after culturing with indoles for 24 h in 96-well plates. *Error bars* indicate SDs. For confocal laser microscope analysis, biofilm formation by *E. coli* K-12 BW25113 (**d**) and *S. aureus* (MSSA) 6538 (**e**) was observed in 96-well plates in the presence and absence of 5-iodoindole. The *scale bars* represent 100 μm. The experiment was performed in triplicate

Since biofilm formation is a major mechanism of bacterial persistence (Costerton et al. 1999) and slow-growing biofilms produce substantial numbers (~1%) of persisters (Lewis 2008), we investigated the effects of 5-iodoindole and of other two halogenated indoles on biofilm formation by *E. coli* and *S. aureus*. Of the indoles tested that reduced persister survival, 5-iodoindole most effectively inhibited biofilm formation by *E. coli* and *S. aureus* and did so in a dose-dependent manner (Fig. 4a, b). For example, 5-iodoindole at 0.2 mM (1/10 of MIC) reduced biofilm formation by *E. coli* (88%) and *S. aureus* (81%). In contrast, 4-fluoroindole and 7-chloroindole, at low levels (0.05–0.2 mM) increased biofilm formation by *E. coli* (Fig. 4a), which concurs with previous observations that sub-inhibitory concentrations of antibiotics induce biofilm formation by *E. coli*, *P. aeruginosa*, and *Staphylococcus epidermidis* (Hoffman et al. 2005; Linares et al. 2006; Rachid et al. 2000). In addition, three halogenated indoles did not affect biofilm formation by *P. aeruginosa* at concentrations up to 2 mM (Fig. 4c).

Since bacteria form biofilms on the bottoms and sides of plates, confocal laser microscopy was used to observe biofilm formation on the bottoms of 96-well polystyrene plates. In-line with our quantitative data, 5-iodoindole at 0.2 mM (1/10 of MIC) dramatically inhibited biofilm formation by *E. coli* and *S. aureus* (Fig. 4d, e). Also, relative amounts of biofilm formation by *E. coli* and *S. aureus* confirmed that *S. aureus* 6538 produced markedly more biofilm than *E. coli* BW25113 under the same conditions.

Biofilm inhibition was further confirmed by COMSTAT analysis as 5-iodoindole was found to reduce all three measured parameters (biomass, mean thickness, and substratum coverage) of *E. coli* and *S. aureus* (Table 2). Specifically, the biomasses (volume/area) of *E. coli* and *S. aureus* biofilms were reduced by 0.2 mM 5-iodoindole by more than 93 and 59%, respectively. Generally, cells in the stationary stage or biofilms contain a high population of persisters more than cells in exponential stage. Therefore, these results show 5-iodoindole effectively kills persisters at exponential and stationary stages, and thus, inhibits biofilm formation.

**Table 2:** COMSTAT analysis results for *E. coli* K-12 BW25113 and *S. aureus* MSSA 6538 biofilms on 96-well plates in the presence of 5-iodoindole

Strain	5-Iodoindole (mM)	Volume/area (μm^3^ μm^−2^)	Mean thickness (μm)	Substratum coverage (%)
*E. coli* K-12 BW25113	None	2.3 ± 0.6	2.9 ± 0.7	26 ± 7
	0.2	0.15 ± 0.03	0.20 ± 0.04	1.4 ± 0.2
*S. aureus* MSSA 6538	None	12.6 ± 0.3	11.6 ± 0.3	98 ± 1
	0.2	5.1 ± 0.4	5.2 ± 0.3	51 ± 6
	0.5	1.8 ± 0.8	2.1 ± 0.9	22 ± 8

### 5-Iodoindole rapidly kills *E. coli* and *S. aureus* without forming persister cells

**Fig. 5:**
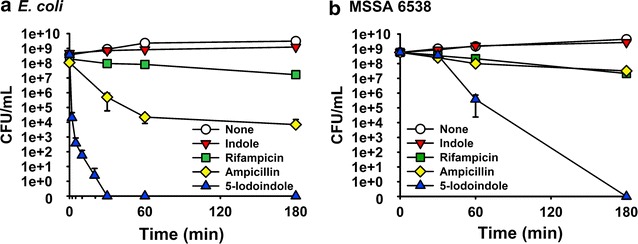
5-Iodoindole does not induce *E. coli* and *S. aureus* persister cell formation. Exponential-phase cultures of *E. coli* K-12 BW25113 (**a**) or *S. aureus* (MSSA) 6538 (**b**) were exposed to indole (2 mM), rifampicin (100 μg mL^−1^), ampicillin (100 μg mL^−1^), or 5-iodoindole (2 mM) at 37 °C, 250 rpm. Cell viabilities were then determined. The experiment was performed in triplicate

In addition to the persister cell killing assay, the effects of 5-iodoindole on persister formation were measured without antibiotic pretreatment. As expected (Kwan et al. 2013), the antibiotics ampicillin or rifampicin at high concentrations (100 μg mL^−1^, 10 × MIC) produced substantial numbers (1–0.01%) of persister cells of *E. coli* and *S. aureus* (Fig. 5a, b). However, 5-iodoindole at 2 mM (~1 × MIC) completely killed all *E. coli* and *S. aureus* cells within 3 h indicating that 5-iodoindole do not produce persister cells (Fig. 5a, b). In the case of *E. coli*, 5-iodoindole eradicated all *E. coli* cells within 30 min. In contrast, indole (the control) at 2 mM did not kill *E. coli* or *S. aureus*. Hence, these results suggest that killing mechanisms of 5-iodoindole and indole, ampicillin, or rifampicin differ, which should be further investigated.

In order to understand how 5-iodoindole kills persister cells, two other iodo compounds (sodium iodide and potassium iodide) were tested. However, neither compound killed persister cells at all at 2 mM (data not shown). This result shows the iodide ion is not a major factor, but that the iodine within the indole moiety reduces persister survival and imparts antimicrobial activity.

### 5-Iodoindole reduces staphyloxanthin production in *S. aureus* strains

**Fig. 6:**
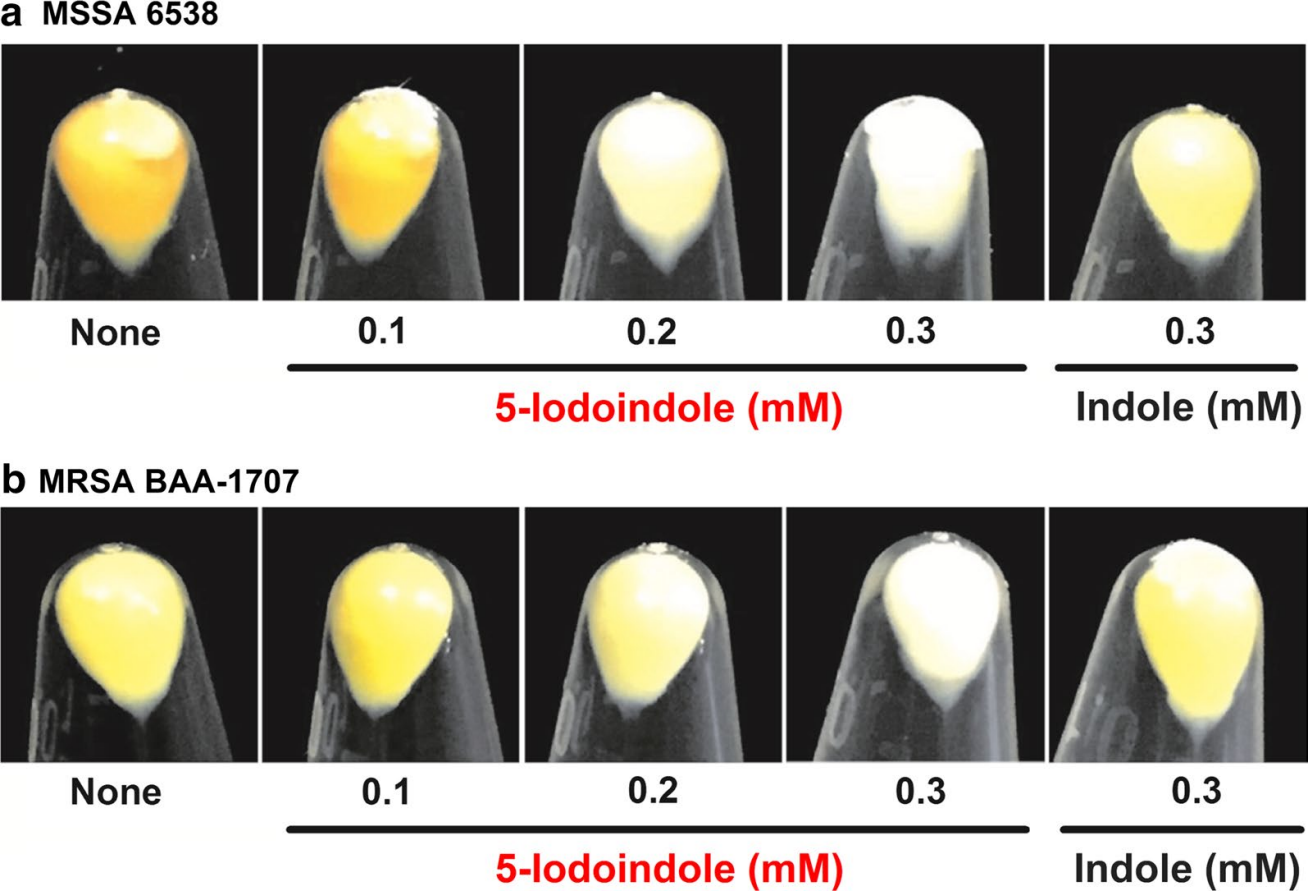
5-Iodoindole inhibits staphyloxanthin production in *S. aureus*. Staphyloxanthin production was determined in the cell pellets of *S. aureus* (MSSA) 6538 (**a**) or a methicillin-resistant *S. aureus* (MRSA) ATCC BAA-1707 (MW 2) (**b**) grown for 24-h in the presence or absence of 5-iodoindole or indole. The experiment was performed in duplicate and representative images are shown

Since indole and 7-benzyloxyindole were found to inhibit the production of staphyloxanthin in *S. aureus* (Lee et al. 2013), we investigated staphyloxanthin production in two *S. aureus* strains (MSSA 6538 and MRSA ATCC BAA-1707). 5-Iodoindole was observed to decrease staphyloxanthin production in a dose-dependent manner (Fig. 6). More specifically, 5-iodoindole at 0.3 mM (1/10 of MIC) abolished staphyloxanthin production and produced colorless cell pellets of two *S. aureus* strains. Furthermore, 5-iodoindole was much more potent than indole. Hence, 5-iodoindole also diminishes the virulence of *S. aureus* strains, including a MRSA strain.

### 5-Iodoindole does not alter *E. coli* and *S. aureus* cell morphology

**Fig. 7:**
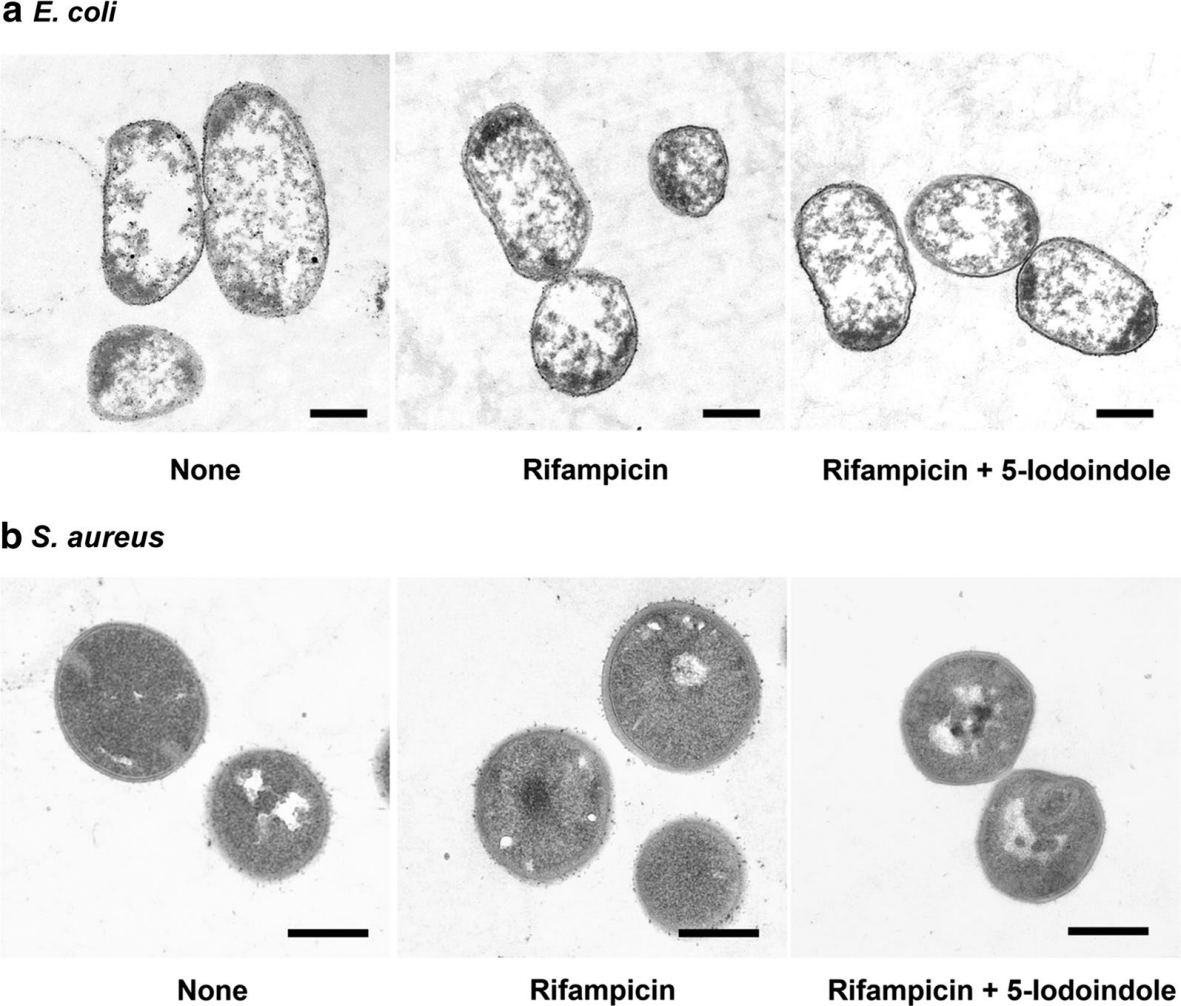
5-Iodoindole does not affect cell morphology. Exponential-phase cultures of *E. coli* K-12 BW25113 (**a**) or *S. aureus* (MSSA) 6538 (**b**) were exposure to rifampicin (100 μg mL^−1^) for 30 min and then incubated with 5-iodoindole (2 mM) or DMSO for 1 h then visualized by transmission electron microscopy. The *scale bars* represent 500 nm

Transmission electron microscopy (TEM) was used to investigate if any morphological changes are induced by 5-iodoindole. Intriguingly, neither rifampicin (100 μg mL^−1^) nor 5-iodoindole (2 mM) caused any obvious cell shape or membrane change of *E. coli* or *S. aureus* (Fig. 7a, b). In the case of rifampicin, this observation was expected because it suppresses cell growth by inhibiting mRNA synthesis (Calvori et al. 1965), and thus, we considered 5-iodoindole might also inhibit mRNA synthesis. To investigate this notion, we isolated the total mRNA of *E. coli* BW25113 cells treated with or without indole or 5-iodoindole treatment for 0, 0.5, and 1 h. However, neither indole nor 5-iodoindole at concentrations up to 2 mM changed the amount or purity of isolated mRNA. For example, the treatment of indole or 5-iodoindole at 2 mM for 1 h produced 460 ± 80 ng mL^−1^ (2.11 at A_260/280_) or 360 ± 80 ng mL^−1^ (2.10 at A_260/A280_) of total mRNA, respectively, while no treatment produced 540 ± 90 ng mL^−1^ (2.12 at A_260/A280_). Hence, it appears that unlike rifampicin, 5-iodoindole does not interfere mRNA synthesis.

## Discussion

The present study was undertaken to identify indole derivatives capable of inhibiting Gram-negative *E. coli* and Gram-positive *S. aureus* persisters and biofilm formation by these two bacteria. Our results show that the different functional groups of indoles differentially controlled *E. coli* persistence, and that several halogenated indoles, especially 5-iodoindole, potently inhibit the formation of persister cells and biofilms by *E. coli* and *S. aureus*, but not those of *P. aeruginosa*. In addition, 5-iodoindole was found to have anti-microbial activities against *E. coli* and *S. aureus*, but unlike other antibiotics, 5-iodoindole does not induce *E. coli* and *S. aureus* persister cell formation. Furthermore, 5-iodoindole reduces the production of the virulence factor staphyloxanthin by *S. aureus*.

In regard to the chemical structures, the addition of halogen atoms to the indole structure, such as, in 4-fluoroindole, 7-chloroindole, 7-bromoindole, 5-iodoindole, and 5-(trifluoromethoxy)indoline-2,3-dione, was found to markedly reduce persister cell survival (Fig. 1). Elemental halogens are potentially toxic and have high reactivities, and both chlorine and bromine are used as disinfectants for drinking water, swimming pools, wounds, spas, dishes, and surfaces. Iodine is the least reactive of the four common halogens, and the biological toxicity of 5-iodoindole has not been studied previously and warrants additional scrutiny.

Currently, studies have described a number of strategies to combat persister cells, such as, (1) killing persister cells in the dormant state, (2) waking persisters and then applying antibiotics, and (3) preventing the formation of persister cells (Wood 2016). Our results indicate 5-iodoindole can prevent persister formation (Fig. 5) and kill persisters in the stationary phase (Figs. 2, 3), and that it can effectively prevent biofilm formation (Fig. 4). Hence, 5-iodoindole is a potent anti-persister compound in that it can both kill and prevent persister cell formation as well as is effective in biofilms.

Although the substituted indoles effectively killed persister cells of *E. coli* and *S. aureus,* including a MRSA strain, they did not effectively kill *P. aeruginosa* persisters (Fig. 3b) or prevent its formation of biofilms (Fig. 4c). Indole stimulates biofilm formation by *P. aeruginosa* (Lee et al. 2007) and diminishes *P. aeruginosa* virulence (Lee et al. 2009). For the halogenated indoles (4-fluoroindole, 7-chloroindole, 7-bromoindole, and 5-iodoindole), *P. aeruginosa* had MICs one magnitude higher than *E. coli* and *S. aureus* (Table 1), but these indoles did not substantially influence biofilm formation (Fig. 4c) or the production of virulence factors (data not shown). Several authors have reported *P. aeruginosa* can resist halogens, such as, chlorine (Seyfried and Fraser 1980), iodine (Brown and Gauthier 1993), and brominated compounds by utilizing its efflux pump (Maeda et al. 2012). Hence, it appears that unlike *E. coli* and *S. aureus*, *P. aeruginosa* has probably developed several resistant mechanisms against halogenated compounds.

Since antibiotics work in different ways, the genetic mechanism responsible for their promotion of persister formation remains elusive, although toxin/antitoxin systems appear to play a role in persister formation (Lewis 2008; Wang and Wood 2011). Several recent reports (Kwan et al. 2013; Conlon et al. 2013; Marques et al. 2014) have suggested protein synthesis (translation) or protein degradation underlie the inhibition of persister cell formation. In the present study, 5-iodoindole did not alter the amount of total mRNA or distinctively change the membrane morphologies of *E. coli* or *S. aureus* (Fig. 7). Thus, we speculate that perturbations of protein synthesis, stability, or protein degradation are responsible, and suggest this possibility be further investigated. Furthermore, our observations indicate that 5-iodoindole could be used in combination with commercial antibiotics to eradicate persister cells and biofilms.

This is the first report to show that halogen-containing indoles should be considered potential agents for the control of persister cells and biofilms. Since hundreds of indole derivatives are commercially available and new indole derivatives are readily constructed (Bunders et al. 2011), further screening of larger libraries of indole derivatives might generate more potent agents for the eradication of human pathogens like pathogenic *E. coli* and *S. aureus*.
